# Supplementary material for: Identification of photoperiod-induced specific miRNAs in the adrenal glands of Sunite sheep (Ovis aries)
Source: Front Vet Sci. 2022 Jul 22;9:888207. doi: 10.3389/fvets.2022.888207 (PMC9354845; doi:10.3389/fvets.2022.888207)
Supplement: Supplementary file 1 [file Data_Sheet_1.ZIP › Supplementary file/miRDeep2 file.zip!/miRDeep2 file/Know_Structure/LP42A1/oar-let-7b.pdf]

miRBase precursor : oar-let-7b
Total read count : 230697
oar-let-7b read count : 229600
remaining reads : 1097

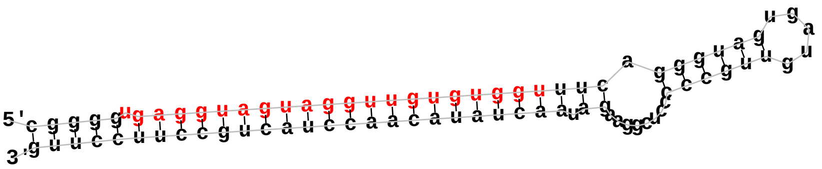

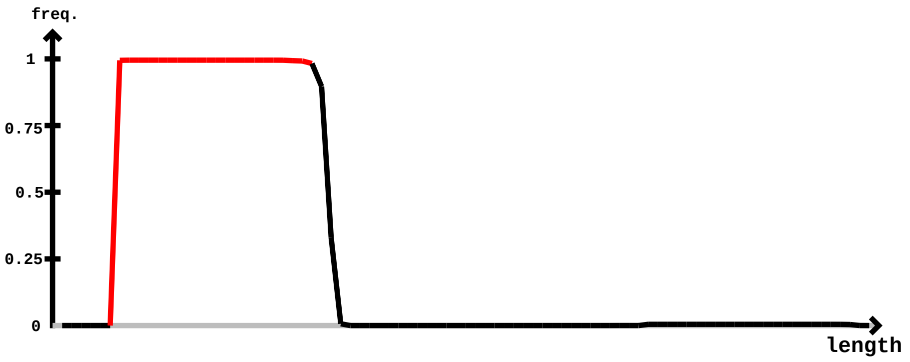


```
       oar-let-7b
5'- cggggugagguaguagguugugugguuucaggguagugauguugcccccucggaagauaacuauacaaccuacugccuuccuug   -3'    exp
    (((((.(((((((((((((((((((((((.((((((.....))))))(....)..)).))))))))))))))))))))))))))    reads     mm        sample
    ....gugagguaguagguugugugguu.........................................................    1         0         seq
    ....gugagguaguagguugugugguuu........................................................    1         0         seq
    .....ugaCguaguagguugug..............................................................    1         1         seq
    .....ugagguaguagguugug..............................................................    48        0         seq
    .....ugagguaguagguugugu.............................................................    395       0         seq
    .....ugagguaguagguugugug............................................................    297       0         seq
    .....ugagguaguagguugugugg...........................................................    2150      0         seq
    .....ugagguaguagguuguguggu..........................................................    19821     0         seq
    .....ugagguaguagguugugugguu.........................................................    130727    0         seq
    .....ugagguaguagguugugugguuu........................................................    74462     0         seq
    .....ugagguaguagguugugugguuuc.......................................................    1534      0         seq
    .....ugagguaguagguugugugguuuca......................................................    5         0         seq
    ......gagguaguagguugugu.............................................................    1         0         seq
    ......gagguaguagguugugugg...........................................................    1         0         seq
    ......gagguaguagguuguguggu..........................................................    13        0         seq
    ......gagguaguagguugugugguu.........................................................    90        0         seq
    ......gagguaguagguugugugguuu........................................................    50        0         seq
    ......gagguaguagguugugugguuuc.......................................................    1         0         seq
    .........guaguagguugugugguuu........................................................    1         0         seq
    ...........aguagguugugugguuu........................................................    1         0         seq
    .............uagguugugugguuucaggguagu...............................................    1         0         seq
    ...........................ucaggguagugauguugc.......................................    1         0         seq
    ...........................ucaggguagugauguugcccccucg................................    1         0         seq
    ...........................ucaggguagugauguugcccccucgga..............................    1         0         seq
    ...........................ucaggguagugauguugcccccucggaagau..........................    1         0         seq
    ...........................ucaggguagugauguugcccccucggaagaua.........................    1         0         seq
    ............................................cccccucggaagauaacuaua...................    1         0         seq
    ............................................cccccucggaagauaacuauac..................    1         0         seq
    ..............................................cccucggaagauaacuauacaacc..............    1         0         seq
    .................................................ucggaagauaacuauacaaccua............    1         0         seq
    ......................................................agauaacuauacaaccuacug.........    1         0         seq
    ......................................................agauaacuauacaaccuacugcc.......    1         0         seq
    ......................................................agauaacuauacaaccuacugccu......    1         0         seq
    ......................................................agauaacuauacaaccuacugccuu.....    1         0         seq
    .........................................................uaacuauacaaccuacugccuu.....    1         0         seq
```

oar-let-7b

```
cggggugagguaguagguuguguggu uucaggguagugauguugcccccucggaagauaacuauacaaccuacugccuuccuug
.........................................................uaacuauacaaccuacugccuuc....    1      0      seq
..........................................................aacuauacaaccuacugccuuc....    1      0      seq
............................................................cuauacaaccuacugccuu.....    2      0      seq
............................................................cuauacaaccuacugccuuc....    7      0      seq
............................................................cuauacaaccuacugccuucc...    152    0      seq
............................................................cuauacaaccuacugccuuccu..    871    0      seq
............................................................cuauacaaccuacugccuuccuu.    41     0      seq
............................................................cuauacaaccuacugccuuccuug    1      0      seq
.............................................................uauacaaccuacugccuucc...    1      0      seq
.............................................................uauacaaccuacugccuuccu..    5      0      seq
```
